# Applicability of liquid biopsies to represent the mutational profile of tumor tissue from different cancer entities

**DOI:** 10.1038/s41388-021-01928-w

**Published:** 2021-07-06

**Authors:** Sandra Liebs, Theresa Eder, Frederick Klauschen, Moritz Schütte, Marie-Laure Yaspo, Ulrich Keilholz, Ingeborg Tinhofer, Evelyn Kidess-Sigal, Diana Braunholz

**Affiliations:** 1 German Cancer Consortium (DKTK), German Cancer Research Center (DKFZ), Heidelberg, Germany; 2 Charité Comprehensive Cancer Center, Charité – Universitätsmedizin Berlin, corporate member of Freie Universität Berlin, Humboldt-Universität zu Berlin, and Berlin Institute of Health, Berlin, Germany; 3 Department of Radiooncology and Radiotherapy, Charité – Universitätsmedizin Berlin, corporate member of Freie Universität Berlin, Humboldt-Universität zu Berlin, and Berlin Institute of Health, Berlin, Germany; 4 Institute of Pathology, Charité – Universitätsmedizin Berlin, corporate member of Freie Universität Berlin, Humboldt-Universität zu Berlin, and Berlin Institute of Health, Berlin, Germany; 5 Alacris Theranostics GmbH, Berlin, Germany; 6 Department of Vertebrate Genomics/Otto Warburg Laboratory Gene Regulation and Systems Biology of Cancer, Max Planck Institute for Molecular Genetics, Berlin, Germany; 7 Department of Medicine, Division of Hepatology and Gastroenterology, Charité – Universitätsmedizin Berlin, corporate member of Freie Universität Berlin, Humboldt-Universität zu Berlin, and Berlin Institute of Health, Berlin, Germany; 8 Berlin Institute of Health (BIH), Berlin, Germany

**Keywords:** Metastasis, Tumour biomarkers

## Abstract

Genetic investigation of tumor heterogeneity and clonal evolution in solid cancers could be assisted by the analysis of liquid biopsies. However, tumors of various entities might release different quantities of circulating tumor cells (CTCs) and cell-free DNA (cfDNA) into the bloodstream, potentially limiting the diagnostic potential of liquid biopsy in distinct tumor histologies. Patients with advanced colorectal cancer (CRC), head and neck squamous cell carcinoma (HNSCC), and melanoma (MEL) were enrolled in the study, representing tumors with different metastatic patterns. Mutation profiles of cfDNA, CTCs, and tumor tissue were assessed by panel sequencing, targeting 327 cancer-related genes. In total, 30 tissue, 18 cfDNA, and 7 CTC samples from 18 patients were sequenced. Best concordance between the mutation profile of tissue and cfDNA was achieved in CRC and MEL, possibly due to the remarkable heterogeneity of HNSCC (63%, 55% and 11%, respectively). Concordance especially depended on the amount of cfDNA used for library preparation. While 21 of 27 (78%) tissue mutations were retrieved in high-input cfDNA samples (30–100 ng, *N* = 8), only 4 of 65 (6%) could be detected in low-input samples (<30 ng, *N* = 10). CTCs were detected in 13 of 18 patients (72%). However, downstream analysis was limited by poor DNA quality, allowing targeted sequencing of only seven CTC samples isolated from four patients. Only one CTC sample reflected the mutation profile of the respective tumor. Private mutations, which were detected in CTCs but not in tissue, suggested the presence of rare subclones. Our pilot study demonstrated superiority of cfDNA- compared to CTC-based mutation profiling. It was further shown that CTCs may serve as additional means to detect rare subclones possibly involved in treatment resistance. Both findings require validation in a larger patient cohort.

## Introduction

Tumor heterogeneity is a major driver of treatment failure in cancer management [1–3]. Genetic, epigenetic, and phenotypic differences between distinct subpopulations of cells within the same tumor lesion may foster a survival benefit for resistant subclones, resulting in primary or secondary resistance [4]. Serial analysis of spatial and temporal heterogeneity within a single lesion and between multiple tumor sites has been suggested to improve in-depth disease monitoring during systemic treatment [5, 6]. To circumvent the invasive procedure of tissue sampling and to overcome its limitations in depicting the highly dynamic genetic complexity of a tumor, the analysis of blood-based biomarkers (liquid biopsy, LB) might increase therapeutic precision. Uncertainty exists concerning the diagnostic information contained in different components of peripheral blood. Circulating tumor cells (CTCs) represent cells disseminating from the tumor tissue, which potentially initiate the formation of metastasis [7–9]. Cell-free DNA (cfDNA) is mainly released from apoptotic and necrotic cells [10]. Despite remaining technological limitations in detection and characterization of cfDNA and CTCs, there is emerging evidence that the analysis of both constituents might allow disease surveillance and therapy guidance [11–13]. Increased CTC numbers and cfDNA concentrations were demonstrated to be of prognostic and predictive value in various tumor entities [14, 15]. Diagnostic applications and longitudinal monitoring of treatment response were mainly based on mutation profiling of LB [13, 16, 17]. Several studies indicated complementarity of CTCs and cfDNA [18, 19], increasing the potential benefit of LB-based patient monitoring based on a single blood draw. The diagnostic potential of CTCs and cfDNA should depend on tumor features including its anatomic location, growth kinetics, invasiveness, and routes of metastatic spread [20, 21], and could thus differ between distinct tumor histologies. To elucidate the ability of liquid biopsies to depict mutations in solid cancers with different metastatic routes, patients with head and neck squamous cell carcinoma (HNSCC), colorectal cancer (CRC), and melanoma (MEL) were enrolled in this study, and the mutation repertoire of primary and/or metastatic tumor tissue was compared with those of cfDNA and CTCs. The three tumor entities were selected based on their different metastatic patterns, as HNSCC is clinically characterized by a predominance of locoregional disease progression, CRC by primarily hepatic metastasis through the portal vein, and MEL by frequent systemic hematogenous spread.

## Results

### Characteristics of the patient cohort

The study enrolled a total of 18 patients with metastatic HNSCC, CRC, or MEL (six each) at a time when high CTC counts and cfDNA levels were expected based on the underlying cancer progression or no previous or ongoing cancer therapy. Two patients had not received any treatment prior blood collection. Patients who had already undergone radio-, chemo-, immuno-, and/or targeted therapy had either a break from treatment (≥1.5 months) or were progressing under therapy at the time when LB was collected. Archival tissue samples were collected between 2010 and 2017, whereas blood samples were drawn in 2017. Tissue of distant metastasis or secondary cancer was available from 17 of 18 patients (94%). Except from four patients, metastatic tissue and LB collection was on the same day (*N* = 5) or after 3–16 weeks (*N* = 9). Sufficient material for paired analysis of primary and metastatic tumor tissue using next-generation sequencing (NGS) was available from eight patients (44%), allowing identification of persistent mutations in contrast to cancer plasticity under treatment pressure. A detailed summary of patient and sample characteristics is provided in Supplementary Tables 1 and 2.

### Liquid biopsies

**Fig. 1:**
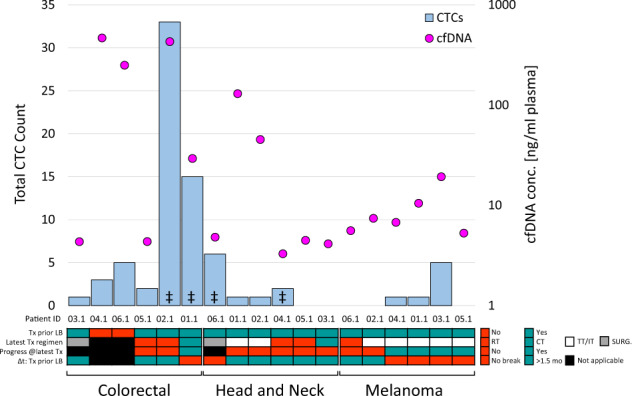
Patient characteristics in comparison to the corresponding cfDNA concentrations and total CTC counts. For each patient, the detectable CTC count and cfDNA concentration were examined, possibly affected by the therapy status, including treatment prior to study enrollment and the time span (Δ*t*) between the last therapy (Tx) and liquid biopsy collection (LB). CT chemotherapy, IT immunotherapy, RT radiotherapy, SURG surgery, TT targeted therapy, mo months. ^‡^available NGS data from whole genome amplified CTCs.

The median concentration of isolated cfDNA per milliliter plasma was 139.7 ng/ml (4.4–468 ng/ml), 4.7 ng/ml (3.3–130 ng/ml), and 7.1 ng/ml (5.3–19.3 ng/ml) in CRC, HNSCC, and MEL patients, respectively. In an exploratory analysis, high cfDNA concentrations were associated with shorter overall survival (OS) of CRC and MEL patients (≥29.4 and ≥10.5 ng/ml with OS ≤ 5 months, respectively), whereas the opposite was observed in the HNSCC cohort (Supplementary Table 1). No correlation between cfDNA concentrations and CTC counts was found. CTCs were detected in 13 of 18 patients (72%). Except for two CRC patients with 15 and 33 detectable CTCs (CRC01.1 and CRC002.1, respectively), total tumor cell counts ranged between zero and six cells within the entire patient cohort. The presence of three or more CTCs per 7.5 ml blood was associated with worse OS in CRC patients (≤ 5 months, Supplementary Table 1). No association was observed in MEL and HNSCC. CTC counts, cfDNA concentrations, and clinicopathological characteristics of each patient are presented in Fig. 1.

In total, 16 CTC samples were collected from 12 patients. For three patients, CTC enrichment was done in parallel using two different protocols, i.e., single CTCs were not only isolated after RosetteSep-based CD45 depletion but CTCs were also collected together with some remaining leucocytes after Ficoll density gradient centrifugation. PCR-based quality control (QC-PCR) results after whole genome amplification (WGA) suggested sufficient DNA integrity for NGS analysis in only 7 of 16 samples (44%) from four patients (Supplementary Fig. 1).

### Representation of tissue mutations in cfDNA

**Fig. 2:**
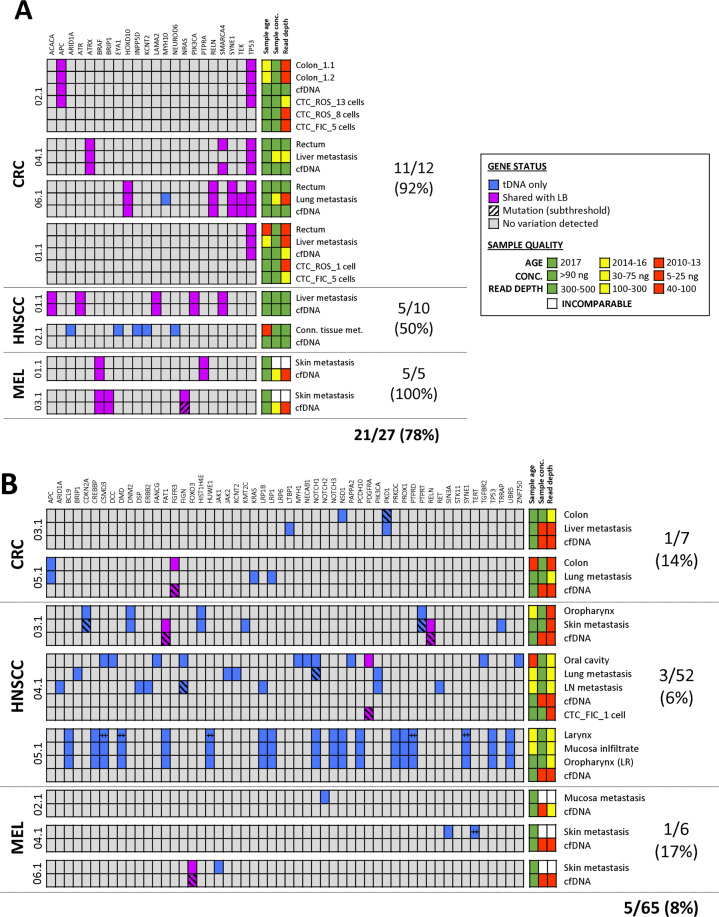
Comparative analysis of tissue-derived mutations and their representation in LB samples. Patients were categorized into two sub-groups referring to **A** high-input and **B** low-input cfDNA samples used for NGS analysis (≥30 and <30 ng, respectively). ^‡^Multiple mutations were detected in the same gene. FIC density gradient centrifugation-enriched CTCs, LB liquid biopsy, LR local recurrence, NGS next-generation sequencing, ROS RosetteSep™-enriched CTCs, tDNA tumor-derived DNA.

In total, solid and LB samples from 18 patients were analyzed, originating from primary and metastatic tumor tissue (*n* = 30), cfDNA (*n* = 18), and CTCs (*n* = 7). Variant calling identified 92 somatic mutations in tissue samples (CRC: 19, HNSCC: 62, MEL: 11), which were examined with regard to their representation in cfDNA. Overall, tissue mutations were detected in 11 of 16 (69%) cfDNA samples (in the remaining two cases, no tissue mutation in the respective panel of genes was identified, leading to the exclusion of those two patients from the analysis of tissue mutation reflection in plasma). Successful retrieval of tissue mutations in plasma depended on the amount of cfDNA used for library preparation and not on the temporal distribution in sample collection. The analysis of high-input cfDNA samples (30–100 ng, *n* = 8) resulted in an overall concordance rate of 78% (CRC: 92%, HNSCC: 50%, MEL: 100%; Fig. 2A). Of the patients for whom the total yield of cfDNA was less than 30 ng (*n* = 8), only 4 of 65 (6%) tissue mutations were also found in plasma (Fig. 2B).

### Representation of tissue mutations in CTCs

In addition to the retrieval of tissue alterations in plasma, CTC samples were analyzed to investigate concordance in mutation profiles with the solid cancer. Limited by the CTC detection rate (72%) and the fraction of samples with sufficient DNA integrity for WGA and sequencing (44%), NGS data were obtained from only seven CTC samples isolated from four patients. In patient HNSCC006.1, the analysis of tumor tissue from the local recurrence in the oral cavity and the lung metastasis did not reveal any mutations. Therefore, assessment of the concordance between tumor tissue and CTCs was limited to patients CRC001.1, CRC002.1, and HNSCC004.1, harboring 1, 2, and 20 tissue alterations, respectively.

For patient CRC002.1, mutations in the tumor suppressor genes *APC* and *TP53* identified in the colon tumor tissue were also found in a pooled sample of 13 CTCs (Fig. 2A). However, the analysis of two additional samples from five and eight CTCs revealed only the wildtype despite comparable DNA integrities (Supplementary Fig. 1). The *TP53* p.Q100* variant detected in the rectum and liver metastasis of patient CRC001.1 was not represented in either of the two available CTC samples (one and five tumor cells) but was detected in the respective cfDNA. Only 1 of 20 tissue mutations identified in patient HNSCC004.1 was verified at subthreshold allele frequency (AF) in the corresponding sample from a single CTC (confirmed to represent a high-quality sample per QC-PCR, Fig. 2B).

### Indication of heterogeneity and clonal evolution in LB

**Fig. 3:**
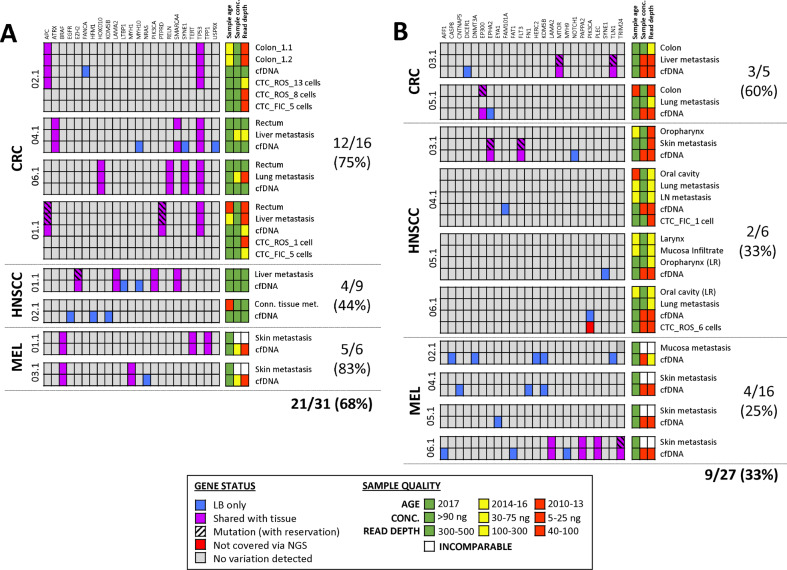
Comparative analysis of cfDNA mutations and their concordance with corresponding tissue samples. Patients were assigned to a sub-group based on **A** high-input and **B** low-input cfDNA samples used for NGS analysis (≥30 and <30 ng, respectively). FIC Ficoll-enriched CTCs, LB liquid biopsy, LR local recurrence, NGS next-generation sequencing, ROS RosetteSep™-enriched CTCs, tDNA tumor-derived DNA.

**Fig. 4:**
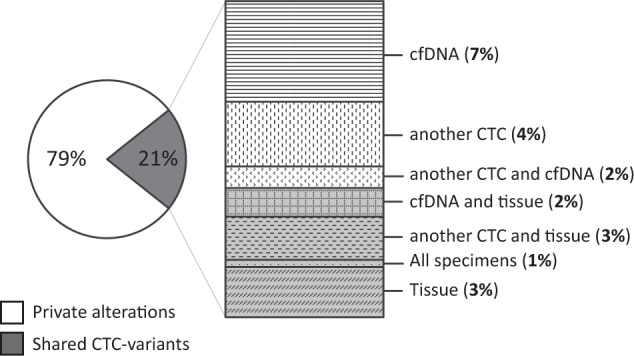
Concordance of CTC-derived alterations with corresponding samples from the same individual. Of a total count of 206 CTC-derived mutations detected in four patients, 44 (21%) were also retrieved in another CTC, cfDNA, and/or tissue sample from the same patient, whereas 162 (79%) were unique for the analyzed circulating tumor cell.

Sequencing results from liquid biopsies were examined for additional alterations, which had not been identified in tissue. We hypothesized that these genetic variations could already be present in solid tumor tissue, though at very low frequencies, and might identify rare subclones that numerically expanded during disease course to the time point of LB collection. In cfDNA, variant calling determined 58 mutations in the entire patient cohort, including 15 tissue mutations (represented in high-input cfDNA samples only). After manual re-analysis (as described in the method section), 15 of 43 (35%) plasma-derived mutations were detected at subthreshold levels in tissue (Fig. 3). CTC samples harbored 206 variants in total, including two predominant tissue mutations (*APC* and *TP53* mutations in the tumor of CRC002.1). Forty-four (21%) CTC mutations were also found in at least one other specimen from the same patient, including another CTC, cfDNA, and/or formalin-fixed paraffin-embedded (FFPE) tissue (Fig. 4). However, only 18 (9%) CTC alterations were also detected in corresponding tumor tissue, out of which 11 (61%) were furthermore retrieved in another LB sample.

### Tumor heterogeneity in patient CRC002.1

Two cancer patients stood out from the entire cohort, highlighting the advantages of LB-based cancer profiling to analyze tumor heterogeneity (CRC002.1) and clonal evolution (MEL003.1). From patient CRC002.1, three CTC pools were sequenced, allowing broader assessment of tumor heterogeneity. Genome integrity indices were comparable between CTC samples with three to four bands in QC-PCR (Supplementary Fig. 1). CTC mutation burden increased with rising cell counts, ranging between 15 and 71 alterations (15 mutations in 5 cells, 36 mutations in 8 cells, and 71 mutations in 13 cells). In contrast, tumor tissue genotyping of two spatial areas of the colon revealed only two mutations in *APC* and *TP53*, which were also detected in the respective cfDNA sample (100 ng input). Only one of three CTC samples (CRC002.1-CTC1, 13 CTCs) reflected the molecular profile of the tissue, whereas the other CTC pools derived from the same patient only displayed the respective wildtype.

With regard to additional variations found in CTC samples, only a small fraction was recovered in other specimens from patient CRC002.1. Sixty-nine mutations were detected in CRC002.1-CTC1, including the two predominant tissue mutations in *APC* and *TP53*. Nine of 67 (13%) CTC mutations were also found at subthreshold AF in the tumor tissue. Tumor heterogeneity was also reflected by the detection of only 8 variants (12%) in another CTC, 7 (10%) in cfDNA, and 1 (1%) in cfDNA and another CTC, whereas 42 alterations (63%) were unique for CRC002.1-CTC1. Consistently, only 9 of 54 mutations (17%) detected in the two other CTC samples from patient CRC002.1 were overlapping with at least one other specimen. The analysis of cfDNA resulted in one additional mutation in the *FANCA* gene, which was not displayed by any other specimen of this patient.

**Fig. 5:**
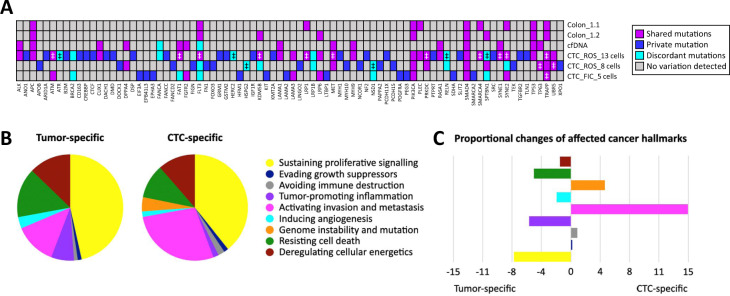
Mutation profiles of corresponding CTCs, cfDNA, and tissue from a single patient reflected tumor heterogeneity, potentially affecting different cancer hallmark-related pathways. **A** Shared, discordant (different mutations in the same gene), and private gene alterations were identified in tumor tissue and LB from CRC002.1 and **B**, **C** assigned to cancer hallmarks, demonstrating distinct differences of involved cancer-related pathways likely to correlate with the requirements concerning tumor growth and metastasis to distant sites. ^‡^Multiple mutations were detected in the same gene.

Kyoto Encyclopedia of Genes and Genomes mapping and further database research (COSMIC, Genetics Home References, National Institutes of Health, PubMed NCBI) was conducted to evaluate pathways possibly impaired by the detected mutations in CTCs and tissue. Pathways were assigned to one or multiple cancer hallmarks, defined by Hanahan and Weinberg [22], and proportional changes between hallmarks specific for the shared mutations of solid tumor tissue and CTC as well as those only found in CTCs were evaluated. It was demonstrated that private CTC mutations were more frequently involved in pathways correlated with invasion, genome instability and avoidance of immune destruction. In contrast, mutations shared by tissue and liquid biopsies were associated with proliferative signaling, tumor-promoting inflammation, resistance to cell death and induction of angiogenesis. A detailed summary of all mutated genes, concordance between specimens, and affected pathways is displayed in Fig. 5.

### Identification of subclonal resistance through LB

**Fig. 6:**
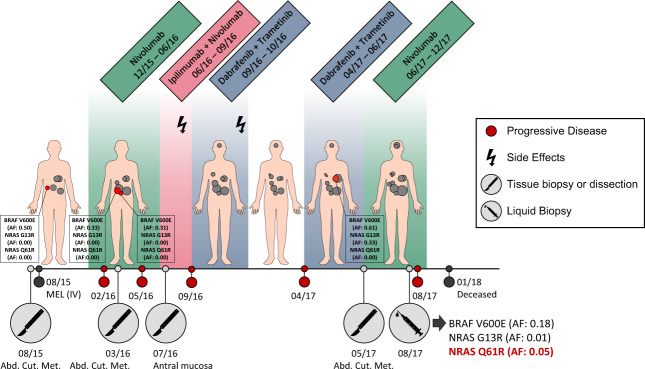
Tissue and plasma genotyping of a patient with refractory melanoma to immunotherapy and BRAF-MEK-inhibition at different time points of the disease. Schematic illustration of the clinical course, including the duration of administered treatment, therapy adaption due to side effects (flash) or progressive disease (red circles), and tumor genotyping conducted on tissue (indicated by a scalpel) or plasma (indicated by a syringe). Allele frequencies (AF) of *BRAF* and *NRAS* mutations were determined by ddPCR analysis.

One patient with refractory MEL to immunotherapy and BRAF-MEK inhibition was of special interest due to the mutation spectrum detected in tissue and cfDNA (Fig. 6 schematically depicts the clinical course of MEL003.1, including genotyping of tissue and plasma). Whole exome sequencing (WES) of a subcutaneous metastasis was performed as part of a precision oncology program of the Charité, revealing the *BRAF* V600E (AF: 0.64) and secondary *NRAS* G13R (AF: 0.41) mutation. After few months of nivolumab treatment, the patient presented with new pulmonary, hepatic and cerebral metastases. At that time, cfDNA displayed the previously reported *BRAF* V600E mutation (AF: 0.26) as well as the *NRAS* G13R mutation at subthreshold level (AF: 0.02). In addition, cfDNA revealed the emergence of the *NRAS* Q61R mutation with an AF of 0.15.

Validation by the highly sensitive Droplet Digital™ PCR (method description in Supplementary information and Supplementary Table 3) confirmed the presence of all three mutations in cfDNA at slightly different AF (V600E: 0.18, G13R: 0.01, Q61R: 0.05). In four metastases resected between 2015 and 2017, *BRAF* V600E was found at AF of 0.31–0.61 in all samples, *NRAS* G13R (AF: 0.33) was only present in one subcutaneous lesion as already known from WES, whereas *NRAS* Q61R was not even detected on subclonal level. All three mutations were proven to be tumor-derived based on their absence in the respective germline sample of the patient (mutant AF: 0.00). Five CTCs were isolated from MEL003.1; however, QC-PCR demonstrated insufficient DNA integrity of cells for sequencing.

## Discussion

Previous studies have suggested superiority of cfDNA as compared to CTC-based mutation profiling [23, 24]. However, mutation detection in cfDNA reflecting the overall profile of cancer cells may differ in informative value from the subset of CTCs, representing a population of cells possibly evading therapy. In this pilot study, we evaluated the feasibility of CTCs and cfDNA in representing the mutational landscape of corresponding tumor tissue in cancer types with distinct metastatic routes. Thus, only patients with advanced disease were enrolled in this study and blood samples were collected shortly after recurrent tumor dissection when highest concordance in the mutation profiles of the solid tumor and LB can be expected.

We observed no difference regarding the informative value of liquid biopsies between tumor entities with distinct metastatic tropism. However, best overall concordance was achieved in CRC followed by MEL, whereas LB-based cancer profiling in HNSCC was less concordant possibly explained by the notoriously heterogeneous mutation profile of this cancer type [25]. A limitation of our study was the small patient cohort, requiring further validation of our observations with a sufficient sample size. In spite of the small number of cases covered per tumor entity, patients were not obviously different from other cohorts with regard to age, sex, clinical course, and metastatic tumor location.

CfDNA outperformed CTC analysis not only with regard to convenience of sample handling, but primarily in reflecting the genomic profile of the solid tissue more closely. Despite the slightly lower cfDNA concentrations isolated in our study compared to previously published data [26–28], tissue mutations were detected in 69% of cfDNA samples. In contrast, Lebofsky et al. found matching mutations in cfDNA and tumor biopsies in 79% of patients with metastatic cancer, recovering 28 of 29 (97%) tissue mutations in plasma [29]. In our study, concordance between tumor tissue and cfDNA was 63%, 55%, and 11% in CRC, MEL, and HNSCC, respectively. In contrast, different groups reported detection of 56–87% of tissue mutations in plasma of CRC patients [20, 26, 30], 73–85% in MEL [17, 31], and 42–92% in HNSCC patients [32, 33]. However, it should be considered that sequencing of high-input cfDNA samples (8 of 18 samples with 30–100 ng) allowed the detection of 21 of 27 (78%) tissue mutations in plasma from our cohort. Thus, concordance rates in highly concentrated cfDNA samples achieved comparable values as previously reported (92% in CRC, 100% in MEL, and 50% in HNSCC). Another interesting exploratory finding was that higher cfDNA concentrations were associated with a shorter OS in CRC and MEL, which is in line with previous results from Bettegowda et al. [20]. The opposite was observed in HNSCC patients; however, this observation must be validated in a bigger patient cohort.

Achieved CTC detection rates were consistent with previous publications or even exceeded reported detection levels (100% of CRC patients: 1–33 CTCs, 67% of HNSCC: 1–6 CTCs, and 50% of MEL: 1–5 CTCs). This might be explained by high tumor aggressiveness in our patient cohort, since ten patients deceased within 6 months after LB collection, whereas only three patients showed an OS of 3.5–4 years to date. In comparison, multiple studies reported detection levels of 20–60% in stage IV CRC patients (1–61 CTCs) [19–21], 41–43% in advanced HNSCC (1 CTC) [15, 34], and 25% in patients with metastatic MEL (≥2 CTCs) [35, 36]. CTC counts were associated with shorter OS in CRC patients, using the cutoff of ≥3 CTCs/7.5 ml [37]. In contrast, application of reported prognostic CTC counts of ≥2 CTCs/7.5 ml blood did not show any association with OS in MEL [35] and HNSCC [15] patients. However, these analyses are exploratory in nature due to the limited sample size and only validation with a larger patient cohort would allow interpretation of this preliminary observation.

The fact that in a significant portion of patients no or only few CTCs were detected might be explained by the limited set of markers applied to identify tumor cells in the peripheral circulation. Different CTC phenotypes express a subset of proteins on their surfaces, hampering the isolation of epithelial, mesenchymal, and hybrid phenotypes of CTCs [38, 39]. Sequencing of solely living CTCs further limited the approach to extracellular markers. Low frequency and quantity of detectable CTCs may limit their diagnostic potential in clinical practice. Even if the analysis of those cells might increase our understanding of the mechanisms of metastatic spread, only 0.01% of CTCs are reported to harbor the potential for colonization in a secondary organ [9], which in turn might contribute to the low concordance between CTCs and metastatic tissue in our study. In addition, technical limitations should not be dismissed when working with single cells. Uniform WGA of CTCs might be impaired by insufficient DNA integrity or allelic imbalance and dropout, reducing the informative value of CTCs for cancer profiling, as seen in our study. Only one sequenced CTC sample reflected the tissue mutation, highlighting the potential of CTCs to reflect tumor heterogeneity, which was particularly evident in patient CRC002.1.

In CRC002.1, tumor heterogeneity was depicted in partially complementary mutation profiles of three CTC samples compared to two spatial areas of the colon tumor tissue and the respective cfDNA sample. Multiple studies determined diversity of mutational status and gene rearrangements in CTCs from the same individual in several cancerous diseases [40–42]. De Luca et al. even reported almost all of the detectable aberrations to be private to each single CTC isolated from breast cancer patients [42]. Interestingly, we demonstrated that gene mutations detected in CTCs were cumulatively assigned to cancer hallmarks matching the requirements of tumor cells circulating in the periphery, including activation of invasion and metastasis [43] as well as avoidance of immune destruction [44]. In contrast, alterations shared by CTCs and tumor tissue were rather associated with requirements of progressing tumor lesions, such as sustaining proliferative signaling, inducing angiogenesis and deregulating cellular energetics [22]. In contrast to cfDNA analysis, only expression profiles of CTCs may provide insight into altered pathways to possibly identify new therapeutically targetable CTC signatures. For example, in the study of Miyamoto et al., CTC heterogeneity in the non-canonical Wnt signaling pathway was linked to resistance against androgen receptor inhibition in a small cohort of prostate cancer patients [45].

In addition, we investigated cancer-clone dynamics in several tumor tissues and the corresponding cfDNA sample from MEL003.1. Here, cfDNA analysis displayed the occurrence of a *NRAS* Q61R mutation after multiple lines of treatment, possibly mediating drug resistance. This case report indicated the pivotal role of clonal evolution under therapeutic pressure and the advantages of LB analysis to detect predominant tissue mutations in plasma when lesions are not accessible for biopsies or only insufficient DNA quantities and/or qualities are available for tissue profiling. Especially in MEL, rapid adaption of the mutational signature in response to selective treatment has been reported [46]. In the study by Gorges et al., continuous changes of the mutation profiles were detected in CTCs from MEL patients regarding the genes *BRAF*, *NRAS*, *EGFR*, and *MAP2K1*. Cancer plasticity during targeted therapy has not only been evident from the analysis of cfDNA and CTCs but also from studies of blood-derived extracellular vesicles as previously published by our group. In the study of Yap et al., a change in the mutant variant profile from *BRAF* V600E to V600K was detected in extracellular vesicles from a MEL patient under BRAF-MEK inhibition, and the emergence of *KRAS* G12D mutation was found after cetuximab treatment of a CRC patient with a *KRAS* wildtype primary tumor [47]. This highlights how promising LB analysis is to detect escape mutations prior to clinical manifestation of cancer progression.

Simultaneous analysis of multiple LB components such as CTCs and cfDNA might improve patient surveillance. Comparable to our results, previous analysis of our group demonstrated an independence of CTC and cfDNA levels in CRC patients (stage I–IV), indicating the great potential for complementary analysis of both fractions [26]. Similarly, synergy was also discussed by Gorges et al., demonstrating that the parallel analysis of CTCs and cfDNA in MEL patients provided supplementary information for monitoring of the underlying disease [46]. This was supported by Onidani et al., who performed NGS analysis of CTCs and cfDNA from patients with HNSCC, CRC, esophageal, and gastric cancer [48]. Low concordance indicated that both biomarkers exhibit private mutation footprints, allowing an increased sensitivity of tissue profiling when analyzing both constituents. Multiple studies reported that 26–52% of variants were solely seen in liquid biopsies [32, 46, 49]. Concerning our pilot project, a possible explanation for the exclusive detection of gene alterations in liquid biopsies but not in tissue is that most of the patients presented with multiple cancer foci, of which only one or two were sequenced and compared to LB. Therefore, analysis of all metastases might reveal increased concordance rates. More importantly, this is again consistent with a high influence of tumor heterogeneity on mutation prevalence in a spatial and temporal manner [3].

From a clinical perspective, adequate diagnostic tools to closely monitor changes in clonal cancer architecture toward disease progression are urgently needed, since most patients develop recurrent or progressive disease despite the many advances in patient management. Therefore, LB should be recognized not as a surrogate for standard tissue profiling but rather as a relevant complementary biomarker to depict the molecular profile of the underlying disease and reveal therapy-induced emergence of cancer subclones. Our results clearly emphasized the advantages of cfDNA-based cancer profiling, indicating a superior utility in CRC and MEL compared to HNSCC. It was furthermore demonstrated that in some patients CTCs may serve as an additional means to detect rare subclones and more closely investigate tumor heterogeneity, possibly leading to treatment resistance. Prior to clinical application, however, standardization of isolation and analysis procedures remains a prerequisite.

## Material and methods

### Patient recruitment and study cohort

Eighteen patients diagnosed with metastasized HNSCC, CRC, and MEL were enrolled in our study at the Charité University Hospital. Patients’ informed written consent was obtained prior to sample collection, which included blood and archival FFPE tissue. Our study was approved by the local ethics committee (EA 4/087/15).

### CTC isolation and whole genome amplification

CTCs were enriched using the RosetteSep™ Human CD45 Depletion kit (Stemcell Technologies, Vancouver, Canada). During the preliminary recruiting phase, an additional blood sample was processed in parallel, from which peripheral blood mononuclear cells were isolated together with CTCs by a density gradient centrifugation protocol using Ficoll-Paque PLUS (GE Healthcare Life Sciences/Merck KGaA, Darmstadt, Germany). CRC- and HNSCC-derived tumor cells were stained for EpCAM and EGFR, whereas MCSP was detected on MEL-CTCs. Leucocytes were identified based on their CD45 expression. In addition, a viability staining was performed using the LIVE/DEAD™ Fixable Blue Dead Cell Stain (Thermo Fisher Scientific, protocol details in Supplementary information). Using Leica’s DMI 3000B inverted microscope for visualization (Leica Biosystems, Wetzlar, Germany), viable CTCs were identified as CD45-negative and tumor marker-positive cells and isolated using the Microinjector IM-9B (Narishige Group, Tokyo, Japan). CTC samples were subjected to an overnight WGA as single or pooled cells according to the manufacturer’s instructions of the REPLI-g Single Cell kit (Qiagen). To evaluate DNA integrity of CTCs and thus effective WGA procedure, a QC-PCR was performed according to the manufacturer’s protocol (Ampli1™ QC kit from Menarini Silicon Biosystems, Castel Maggiore, Italy). The Ampli1™ QC kit amplifies up to four DNA fragments of different size and chromosomal location to predict successful downstream application.

### Library preparation and targeted sequencing

A detailed description of the DNA isolation from whole blood, plasma, and FFPE specimens is given in the Supplementary information. The HaloPlex™ HS target enrichment system for Illumina sequencing (Agilent Technologies, Santa Clara, USA) was used for mutational profiling of archival tumor tissue from CRC and HNSCC patients as well as the LB-derived samples from the entire cohort. Our in-house panel was designed to detect frequently mutated genes of functional relevance in cancer [50], targeting the exonic sequence of 327 genes (1.47 Mb). Library preparation was performed as previously described following manufacturer’s protocol (Agilent, protocol version C1, December 2016) [50]. DNA input varied depending on the available DNA concentrations isolated from different starting material, such as FFPE tissue, whole blood, cfDNA, or CTCs, ranging between 10 and 100 ng. Paired-end sequencing was carried out on the Illumina NextSeq500 platform with the High Output v2 sequencing kit (300 cycles, Illumina, San Diego, USA).

Sequencing of the metastatic tissue of the MEL-cohort was performed as part of the Treat20plus study conducted at the Max Planck Institute in partnership with the Charité Comprehensive Cancer Center. WES was performed on the HiSeq™ system following the protocol of the Nextera Rapid Capture Exome and Expanded Exome kit (Illumina), which covers 201,121 target regions and comprises approximately 62 Mb of DNA.

### Sequencing data analysis and variant calling

Raw fastq files were processed with the Agilent SureCall Software (version 3.5.1.46). A median sequencing depth of 158-fold, 83-fold, 74-fold, and 47-fold was achieved in germline, FFPE, cfDNA, and CTC samples, respectively. Personal alterations were excluded when detected in the individual whole blood sample. Remaining alterations were sieved based on their predicted deleterious effect annotated in the COSMIC database [51, 52] and the Cancer Genome Interpreter [53, 54]. Further analysis was performed as previously described [50]. The detailed procedure of variant calling and data analysis is described in the Supplementary information and depicted in Supplementary Fig. 2. Sequencing data will be available from the corresponding author upon reasonable request.

### Statistical analysis

Continuous variables were summarized by median and range, and categorical variables by frequency. Due to the small sample size, no statistical comparisons were made.

## Supplementary information

Supplementary Figure 1

Supplementary Figure 2

Supplementary information (methods)

Supplementary Table 1

Supplementary Table 2

Supplementary Table 3

Legends of Supp. Figures and Tables
