# Supplementary material for: Applicability of liquid biopsies to represent the mutational profile of tumor tissue from different cancer entities
Source: Oncogene. 2021 Jul 6;40(33):5204–12. doi: 10.1038/s41388-021-01928-w (PMC8376638; doi:10.1038/s41388-021-01928-w)
Supplement: Supplementary file 1 — Supplementary Figure 1 [file 41388_2021_1928_MOESM1_ESM.pdf]

Supplementary Figure 1

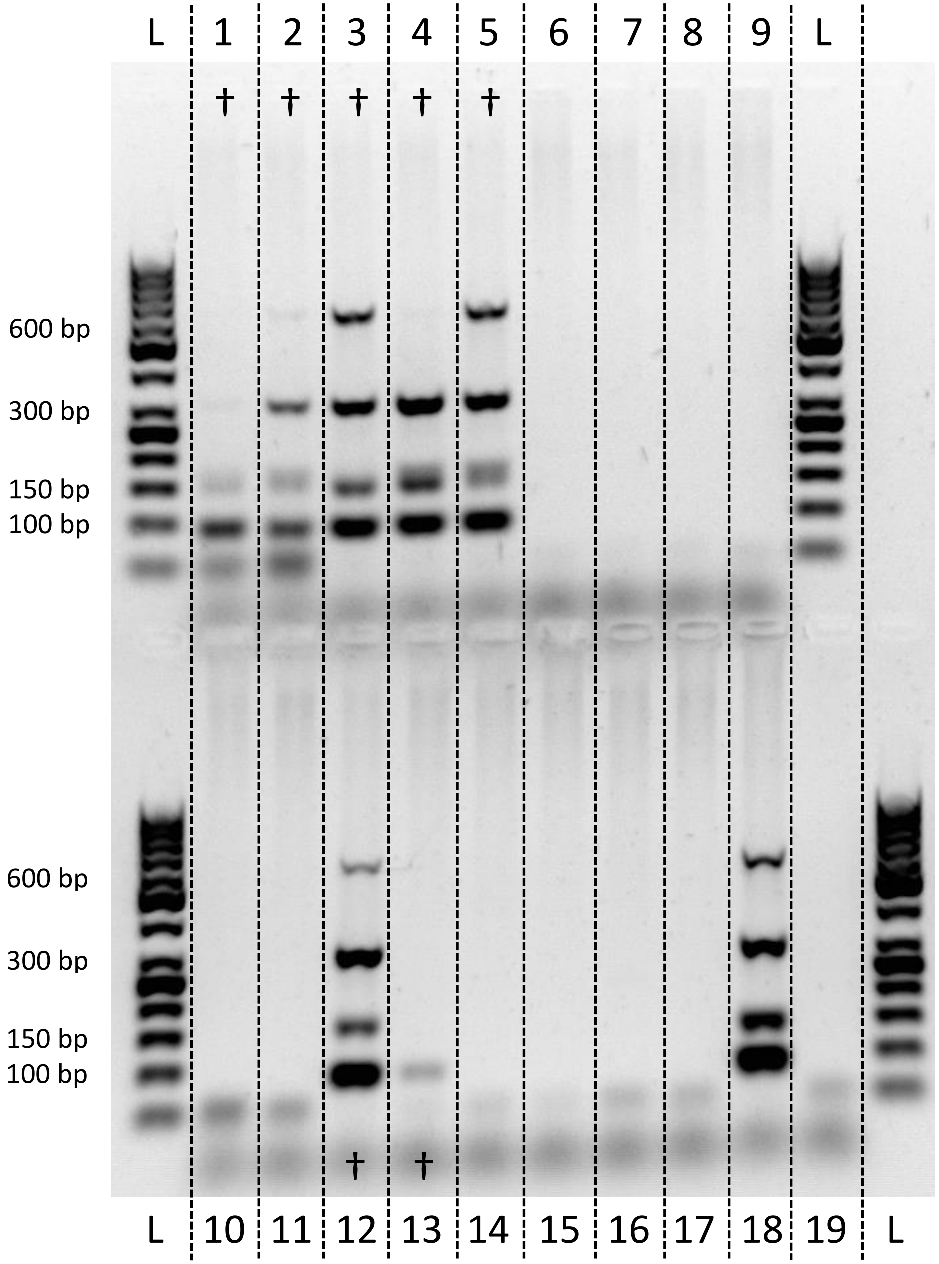

1: CRC001.1_R (1 CTC)
2: CRC001.1_F (5 CTCs)
3: CRC002.1_R (13 CTCs)
4: CRC002.1_R (8 CTCs)
5: CRC002.1_F (5 CTCs)
6: CRC003.1_R (1 CTC)
7: CRC004.1_R (3 CTCs)
8: CRC005.1_R (2 CTCs)
9: CRC006.1_R (5 CTCs)
10: HNSCC002.1_R (1 CTC)
11: HNSCC004.1_R (1 CTC)
12: HNSCC004.1_F (1 CTC)
13: HNSCC006.1_R (6 CTCs)
14: MEL001.1_R (1 CTC)
15: MEL003.1_R (5 CTCs)
16: MEL004.1_R (1 CTC)
17: LS174T cell line (3 cells)
18: Pos. Ctrl (WGA of gDNA)
19: Neg. Ctrl (WGA of H_2O)
