# Supplementary figures and images for: Applicability of liquid biopsies to represent the mutational profile of tumor tissue from different cancer entities
Source: Oncogene. 2021 Jul 6;40(33):5204–12. doi: 10.1038/s41388-021-01928-w (PMC8376638; doi:10.1038/s41388-021-01928-w)

Supplementary Figure 2

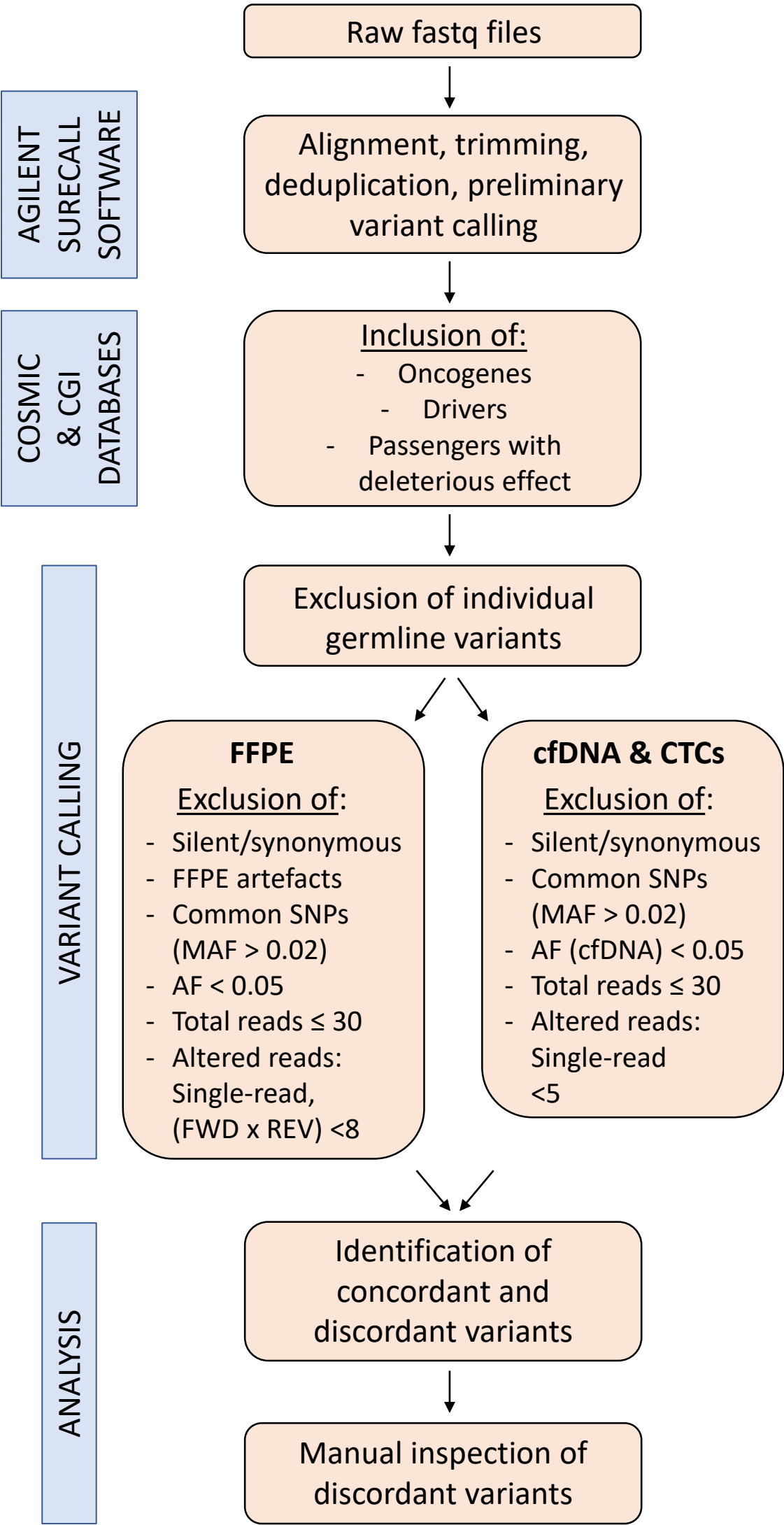

Supplement: Supplementary file 2 — Supplementary Figure 2 [file 41388_2021_1928_MOESM2_ESM.pdf]
