# Supplementary material for: Applicability of liquid biopsies to represent the mutational profile of tumor tissue from different cancer entities
Source: Oncogene. 2021 Jul 6;40(33):5204–12. doi: 10.1038/s41388-021-01928-w (PMC8376638; doi:10.1038/s41388-021-01928-w)
Supplement: Supplementary file 3 — Supplementary information (methods) [file 41388_2021_1928_MOESM3_ESM.docx]

**Supplementary information**

**DNA extraction from whole blood, plasma and FFPE specimens**

Peripheral blood was collected in BD Vacutainer™ K2-EDTA tubes (BD, Franklin Lakes, USA) and processed within two hours of blood sampling. To differentiate tumor-specific somatic mutations from germline and clonal hematopoiesis-related variants, 1-2 ml whole blood was stored at -80°C until further processing. DNA was isolated using the QIAamp DNA Blood Midi kit (Qiagen, Hilden, Germany), applying small adjustments to manufacturer's specifications. Centrifugation during washing steps was performed at 3 200 g for 2 min and 15 min, requiring removal of residual ethanol by incubating at 70°C for 10 min. DNA was eluted in 300 µl Buffer AE twice (3 200 g for 5 min), quantified via UV-Vis absorbance using the Implen NanoPhotometer® P-Class P 330 (Implen, Munich, Germany) and stored at -20°C until further processing.

Plasma for cfDNA isolation was separated via centrifugation at 1 391 g for 7 min and further purified at 2 473 g for 10 min. Until further processing, plasma was stored at -80°C. As specified by the manufacturer, the QIAamp Circulating Nucleic Acid kit (Qiagen) was used for cfDNA isolation from plasma using the QIAvac 24 Plus vacuum manifold. DNA was eluted in 75 µl Buffer AVE twice and concentrated to 50 µl following the manufacturer’s protocol of Zymo’s DNA Clean & Concentrator™-5 kit (Freiburg, Germany).

Ten micrometer thick FFPE slides from primary and metastatic tumor tissue were deparaffinized and processed following the instructions of the High Pure FFPET DNA Isolation kit (Roche Holding AG, Basel, Switzerland). DNA was eluted in 30 µl Elution Buffer. Concentrations of plasma and tissue-derived DNA were determined using the Qubit™ 4 Fluorometer, according to the Qubit® dsDNA HS Assay kit (Thermo Fisher Scientific, Waltham, USA).

**CTC enrichment and staining protocol**

In both CTC enrichment protocols, blood diluted in in phosphate-buffered saline (PBS) supplemented with 2% fetal calf serum (FCS) was centrifuged over Ficoll-Paque PLUS for 35 min at 400 g (with the brake off). The enriched cell fraction was washed in PBS + 2% FCS at 500 g for 15 min. The RosetteSep™ protocol furthermore included a centrifugation step at 120 g for 15 min to remove platelets. Remaining cells were resuspended in 100 µl PBS + 2% FCS and incubated with 10 µl of FcR Blocking Reagent (Miltenyi Biotec, Bergisch Gladbach, Germany) for 10 min followed by a 10 min incubation step with a combination of fluorescence-labelled antibodies. CRC and HNSCC-derived patient samples were incubated with antibodies to EpCAM-AF488 (1 µl, clone 9C4, #324210, BioLegend, San Diego, USA), EGFR-AF488 (1.5 µl, clone AY13, #352908, BioLegend), CD73-FITC (1 µl, clone AD2, #344015, BioLegend) and CD45-AF647 (2 µl, clone HI30, #304020, BioLegend), whereas the MEL-specific tumor marker was detected using anti-MCSP-AF488 (4 µl, clone 9.2.27, #562413, BD). To determine the viability of cells, 2 µl LIVE/DEAD™ Fixable Blue Dead Cell Stain for UV excitation was added for additional 10 min.

**Sequencing data analysis and variant calling**

Analysis of raw fastq files using Agilent`s SureCall Software included sequence alignment to hg19 reference genome, trimming of sequencing adaptors, duplicate removal and preliminary variant calling. COSMIC-annotated, pathogenic mutations as well as CGI-classified oncogenes and predicted drivers were taken into account. Additionally, passenger mutations with a high to very high deleterious effect as well as those with up to a low impact but a predicted drug effect were integrated in our analysis. Silent and synonymous variants were excluded as well as FFPE artefacts and common polymorphisms with a minor allele frequency greater than two percent (as listed in the NCBI dbSNP database). Tissue and cfDNA-derived alterations with an allele frequency of 0.05 or higher were integrated into further analysis, whereas variant calling in CTC pools was not limited by a cut-off value in case of an amplification bias caused by WGA. However, CTC and cfDNA-derived variants with a sequencing depth ≤30x and <5x for total and altered reads, respectively, were not considered. In case of discordant results between tissue and liquid biopsy samples from the same individual, manual inspection revealed few concordant reads, designated as a subthreshold mutation.

**Variant detection using Droplet Digital™ PCR**

PCR reactions contained 1X ddPCR Supermix for Probes with no dUTP (Bio-Rad Laboratories, Hercules, USA), each primer at final concentrations of 900 nM and probe concentrations as listed in Supplementary Table 3. Analysis of cell line-derived gDNA as positive controls furthermore included EcoRI-HF (New England Biolabs, Ipswich, USA) in a final enzyme concentration of 0,5 units/µl. Sample preparation and analysis was performed as previously described [16] and annealing temperatures of each assay were listed in Supplementary Table 3. Patient-derived samples were analyzed in duplicates. Non-template controls as well as positive controls harboring the mutation of interest in front of the background of wild-type DNA were included in every assay.
