# Supplementary material for: Applicability of liquid biopsies to represent the mutational profile of tumor tissue from different cancer entities
Source: Oncogene. 2021 Jul 6;40(33):5204–12. doi: 10.1038/s41388-021-01928-w (PMC8376638; doi:10.1038/s41388-021-01928-w)
Supplement: Supplementary file 4 — Supplementary Table 1 [file 41388_2021_1928_MOESM4_ESM.docx]

| **Patient ID** | **Sex** | **Age** | **Tx prior LB** | **Δt Tx** | **No. of** | **Δt mTB** | **cfDNA** | **Total** | **CTCs/7.5ml** | **Response Eva.** | **OS** |
| --- | --- | --- | --- | --- | --- | --- | --- | --- | --- | --- | --- |
|  |  |  |  | **prior LB** | **met. loci** | **and LB** | **[ng/ml]** | **CTCs** | **blood** | **at LB collection** | **after LB** |
| CRC001.1 | M | 70 | SUR, TT, RT, **CT** | N/A | 2 | 668 d | 29.4 | 15 | 7 | PD (under Tx) | 5 mo |
| CRC002.1 | M | 75 | SUR, CT, TT, **RT** | 4 mo | 4 | 928 d | 430.0 | 33 | 14 | PD (Tx break) | 1 mo |
| CRC003.1 | F | 52 | SUR | N/A | 2 | 52 d | 4.4 | 1 | 1 | No PD | 48 mo |
| CRC004.1 | F | 49 | N/A | N/A | 1 | 20 d | 468.0 | 3 | 3 | at diagnosis | 1 mo |
| CRC005.1 | M | 63 | SUR, CT, **RT** | 3 yrs | 2 | 0 d | 4.4 | 2 | 2 | PD (Tx break) | 48 mo |
| CRC006.1 | M | 68 | N/A | N/A | 3 | 0 d | 250.0 | 5 | 4 | at diagnosis | 4 mo |
| HNSCC001.1 | M | 65 | CT, **TT** | 8 mo | 3 | 1 d | 130.0 | 1 | 0 | PD (Tx break) | 11 mo |
| HNSCC002.1 | M | 74 | RT, CT, **TT** | 11 mo | 6 | 1896 d | 45.4 | 1 | 0 | PD (Tx break) | 17 mo |
| HNSCC003.1 | M | 49 | TT, SUR, **CT** | 4 mo | 6 | 25 d | 4.1 | 0 | 0 | PD (Tx break) | 1 mo |
| HNSCC004.1 | M | 62 | SUR, **RT** | 3 yrs | 4 | 452 d | 3.3 | 2 | 1 | PD (Tx break) | 2 mo |
| HNSCC005.1 | F | 49 | **RT** | 7 mo | 1 | 44 d | 4.5 | 0 | 0 | PD (Tx break) | 6 mo |
| HNSCC006.1 | M | 63 | SUR | N/A | 2 | 27 d | 4.8 | 6 | 6 | PD (after surgery) | 4 mo |
| MEL001.1 | F | 52 | SUR, INF, TT, **IT** | N/A | 4 | 0 d | 10.5 | 1 | 1 | PD (under Tx) | 1 mo |
| MEL002.1 | F | 58 | SUR, RT, INF, CT, IT, **TT** | 1.5 mo | 2 | 23 d | 7.4 | 0 | 0 | PD (Tx break) | 16 mo |
| MEL003.1 | F | 51 | SUR, TT, **IT** | N/A | 9 | 82 d | 19.3 | 5 | 4 | PD (under Tx) | 5 mo |
| MEL004.1 | M | 59 | SUR, RT, IT, **TT** | N/A | 3 | 179 d | 6.6 | 1 | 1 | PD (under Tx) | 10 mo |
| MEL005.1 | M | 64 | SUR, INF, RT, **IT** | N/A | 5 | 122 d | 5.3 | 0 | 0 | PD (under Tx) | 9 mo |
| MEL006.1 | F | 52 | SUR, CT, IT, **RT** | 8 mo | 7 | 0 d | 5.6 | 0 | 0 | PD (Tx break) | 42 mo |

**Supplementary Table 1: Detailed overview of patient characteristics.** Time span (Δt) between the collection of metastatic tissue (mTB) and liquid biopsy (LB) as well as the time span between the last therapy administered (Tx, highlighted in bold) and the LB collection were determined. Prognostic information including number of metastatic loci (no. of met. loci), the response evaluation at the time point (Response Eva. at LB collection) and overall survival of patients after LB collection were included. M: male, F: female, CT: chemotherapy, INF: interferon, IT: immunotherapy, RT: radiotherapy, SUR: surgery, TT: targeted therapy, d: days, mo: months.
