# Supplementary material for: Applicability of liquid biopsies to represent the mutational profile of tumor tissue from different cancer entities
Source: Oncogene. 2021 Jul 6;40(33):5204–12. doi: 10.1038/s41388-021-01928-w (PMC8376638; doi:10.1038/s41388-021-01928-w)
Supplement: Supplementary file 5 — Supplementary Table 2 [file 41388_2021_1928_MOESM5_ESM.docx]

| **Patient ID** | **Primary** | | | **Metastasis / Secondary cancer** | | | **Blood**  **Sampling** | **cfDNA** | **CTC**  **NGS input** |  |
| --- | --- | --- | --- | --- | --- | --- | --- | --- | --- | --- |
|  | **Location** | **Resection** | **NGS input** | **Location** | **Resection** | **NGS**  **input** |  | **NGS input** |  |  |
| CRC001.1 | Rectum | 11/2010 | 100 ng | Liver | 03/2015 | 100 ng | 01/2017 | 94 ng | 100 ng | |
| CRC002.1 | Colon | 08/2014 | 100 ng | [Liver] | [08/2014] | N/A | 02/2017 | 100 ng | 100 ng | |
| CRC003.1 | Colon | 03/2017 | 100 ng | Liver | 02/2017 | 14 ng | 04/2017 | 11 ng |  | |
| CRC004.1 | Rectum | 04/2017 | 100 ng | Liver | 04/2017 | 50 ng | 05/2017 | 100 ng |  | |
| CRC005.1 | Colon | 2012 | 100 ng | Lung | 05/2017 | 100 ng | 05/2017 | 12 ng |  | |
| CRC006.1 | Rectum | 06/2017 | 100 ng | Lung | 07/2017 | 44 ng | 07/2017 | 100 ng |  | |
| HNSCC001.1 | [Tongue] | [2015] | N/A | Liver | 01/2017 | 100 ng | 01/2017 | 100 ng |  | |
| HNSCC002.1 | [Oropharynx] | [09/2010] | N/A | Conn. tissue | 09/2010 | 100 ng | 02/2017 | 92 ng |  | |
| HNSCC003.1 | Oropharynx | 07/2015 | 100 ng | Skin | 02/2017 | 100 ng | 02/2017 | 13 ng |  | |
| HNSCC004.1 | Oral cavity | 12/2013 | 100 ng | Lung + LN | 12/2015 | 50+100 ng | 03/2017 | 7 ng | 100 ng | |
| HNSCC005.1 | Larynx + Infilt. | 08/2016 | 100 ng | Oropharynx | 04/2017 | 100 ng | 05/2017 | 10 ng |  | |
| HNSCC006.1 | Oral cavity‡ | 03/2017 | 100 ng | Lung | 05/2017 | 50 ng | 05/2017 | 15 ng | 100 ng | |
| MEL001.1 | [Skin] | [02/2014] | N/A | Skin | 07/2017 | N/A | 07/2017 | 30 ng |  | |
| MEL002.1 | [Mucosa] | [1999] | N/A | Mucosa | 07/2017 | N/A | 08/2017 | 24 ng |  | |
| MEL003.1 | [Skin] | [07/2015] | N/A | Skin | 05/2017 | N/A | 08/2017 | 50 ng |  | |
| MEL004.1 | [Skin] | [03/2016] | N/A | Skin | 04/2017 | N/A | 10/2017 | 15 ng |  | |
| MEL005.1 | [Mucosa] | [09/2013] | N/A | Skin | 07/2017 | N/A | 11/2017 | 12 ng |  | |
| MEL006.1 | [Skin] | [1995] | N/A | Skin | 11/2017 | N/A | 11/2017 | 16 ng |  | |

**Supplementary Table 2: Detailed overview of tissue and blood specimens.** All cfDNA samples were sequenced, whereas, only from four patients, single CTCs were subjected to library preparation and next generation sequencing due to insufficient amounts of amplified DNA after whole genome amplification in the remaining samples. Tumor tissue in square brackets was not sequenced. Infilt.: Mucosa Infiltrate, LR: local recurrence, Conn. tissue: connective tissue, LN: lymph node, N/A: not applicable. †Available NGS data from 100 ng of whole genome amplified CTC samples, ‡ Instead of the primary tumor from 2013, the local recurrence from 2017 was analyzed from patient HNC006.1.
