# Supplementary material for: Applicability of liquid biopsies to represent the mutational profile of tumor tissue from different cancer entities
Source: Oncogene. 2021 Jul 6;40(33):5204–12. doi: 10.1038/s41388-021-01928-w (PMC8376638; doi:10.1038/s41388-021-01928-w)
Supplement: Supplementary file 6 — Supplementary Table 3 [file 41388_2021_1928_MOESM6_ESM.docx]

**Supplementary Table 3: Duplex ddPCR conditions for variant detection in *BRAF* and *NRAS***

| **Gene** | **Oligo** | **Sequence (5’ – 3’)** | **Final conc.** | **Annealing Temp** |
| --- | --- | --- | --- | --- |
| *BRAF* | FWD | TTTACTTACTACACCTCAGATATA | 900 nM | 56°C |
|  | REV | ACTGTTCAAACTGATGGGA | 900 nM |  |
|  | WT | HEX-TTTGGTCTAGCTACAGTGAAATCTCG-BHQ1 | 200 nM |  |
|  | V600E | FAM-TTTGGTCTAGCTACAGAGAAATCTCG-BHQ1 | 500 nM |  |
| *NRAS* | FWD | GTTCTTGCTGGTGTGAAATG | 900 nM | 61°C |
|  | REV | AGTGGTTCTGGATTAGCTGG | 900 nM |  |
|  | WT | HEX-AGCAGGTGGTGTTGGGAAAAG-BHQ1 | 250 nM |  |
|  | G13R | FAM-AGCAGGTCGTGTTGGGAAAAG-BHQ1 | 250 nM |  |
| *NRAS* | FWD | CAAGTGGTTATAGATGGTGAAACC | 900 nM | 62°C |
|  | REV | CCTTCGCCTGTCCTCATGTATT | 900 nM |  |
|  | WT | HEX-ACTGGATACAGCTGGACAAGAAGAGT-BHQ1 | 250 nM |  |
|  | Q61R | FAM-ACTGGATACAGCTGGACGAGAAGAGT-BHQ1 | 150 nM |  |
