# Supplementary material for: Applicability of liquid biopsies to represent the mutational profile of tumor tissue from different cancer entities
Source: Oncogene. 2021 Jul 6;40(33):5204–12. doi: 10.1038/s41388-021-01928-w (PMC8376638; doi:10.1038/s41388-021-01928-w)
Supplement: Supplementary file 7 — Legends of Supp. Figures and Tables [file 41388_2021_1928_MOESM7_ESM.docx]

**Supplementary Figure 1: Quality control PCR (QC-PCR) of single cell samples to evaluate DNA integrity and successful whole genome amplification for subsequent panel sequencing.** From twelve patients, 16 CTC samples were collected after RosetteSep (R) or Ficoll (F) processing and subjected to WGA. Subsequent Ampli1™ QC-PCR amplified up to four targets of different chromosomal location and amplicon size (91bp, 108-166bp, 299bp and 614bp). †Samples with sufficient amount of amplified DNA from CTCs for successful sequencing. L: 50bp DNA Ladder, LS174T: CRC cell line.

**Supplementary Figure 2: Flow chart demonstrating the data processing algorithm for the identification of high-confidence single nucleotide variants.**

**Supplementary Table 1: Detailed overview of patient characteristics.** Time span (Δt) between the collection of metastatic tissue (mTB) and liquid biopsy (LB) as well as the time span between the last therapy administered (Tx, highlighted in bold) and the LB collection were determined. Prognostic information including number of metastatic loci (no. of met. loci), the response evaluation at the time point (Response Eva. at LB collection) and overall survival of patients after LB collection were included. M: male, F: female, CT: chemotherapy, INF: interferon, IT: immunotherapy, RT: radiotherapy, SUR: surgery, TT: targeted therapy, d: days, mo: months.

**Supplementary Table 2: Detailed overview of tissue and blood specimens.** All cfDNA samples were sequenced, whereas, only from four patients, single CTCs were subjected to library preparation and next generation sequencing due to insufficient amounts of amplified DNA after whole genome amplification in the remaining samples. Tumor tissue in square brackets was not sequenced. Infilt.: Mucosa Infiltrate, LR: local recurrence, Conn. tissue: connective tissue, LN: lymph node, N/A: not applicable. †Available NGS data from 100 ng of whole genome amplified CTC samples, ‡ Instead of the primary tumor from 2013, the local recurrence from 2017 was analyzed from patient HNC006.1.

**Supplementary Table 3: Duplex ddPCR conditions for variant detection in *BRAF* and *NRAS***
